# Treatment of Pelvic Recurrence After Radiotherapy for Cervical Cancer

**DOI:** 10.3390/cancers17243934

**Published:** 2025-12-09

**Authors:** Yanan Song, Kun Zou, Lijuan Zou

**Affiliations:** 1 Department of Radiation Oncology, The Second Affiliated Hospital, Dalian Medical University, Dalian 116021, China; syn0711@163.com; 2 Department of Radiation Oncology, The First Affiliated Hospital, Dalian Medical University, Dalian 116011, China

**Keywords:** cervical cancer, chemotherapy, immunotherapy, systemic therapy, targeted therapy

## Abstract

Pelvic recurrence following radiotherapy for cervical cancer presents a major therapeutic challenge with historically poor prognosis and limited options. This review comprehensively examines the evolving landscape of management strategies for this condition, encompassing both local and systemic approaches. We discuss the roles of salvage surgery and advanced re-irradiation techniques, including stereotactic body radiotherapy and image-guided brachytherapy, highlighting their potential and associated toxicities. A significant focus is placed on the revolution in systemic therapy, particularly the integration of targeted agents—such as anti-angiogenic drugs, PARP inhibitors, and tyrosine kinase inhibitors—and immunotherapy, chiefly immune checkpoint inhibitors like pembrolizumab and cemiplimab. These modalities have demonstrated substantial improvements in survival outcomes in clinical trials. The review underscores the critical shift towards personalized medicine, where treatment selection is increasingly guided by molecular profiling. Finally, we explore future directions, including combination strategies, novel immunotherapies, and emerging technologies, which collectively promise to transform the management of recurrent cervical cancer from palliative control towards the goals of durable remission and functional cure.

## Simple Summary

Despite effective initial treatments, cervical cancer can sometimes recur in the pelvic area, which is very difficult to treat and leaves patients with very few options. This review explores the latest advances in managing this challenging situation. We summarize the potential of new treatment strategies, including modern surgical techniques, advanced forms of radiation, and powerful new drugs like immunotherapies and targeted therapies. Our goal is to provide a clear overview of these emerging options. By compiling this information, we hope to guide doctors toward more personalized and effective treatment plans, ultimately improving survival and quality of life for women facing a recurrence of this disease.

## 1. Introduction

Cervical carcinoma holds the fourth position in global incidence among women, trailing behind lung, colorectal, and breast cancer [1]. The most significant prevalence of new cervical carcinoma incidents is observed in nations characterized by low and middle-income economies [1]. Pelvic recurrence after cervical cancer radiotherapy refers to cervical cancer patients who have undergone radical radiotherapy and achieved complete remission after treatment, but there may be a subsequent occurrence of tumor proliferation at the initial site, which could include the cervix, parametrium, or vagina, as well as in the pelvic lymph nodes, with the malignancy remaining localized within the pelvic area and absent any metastasis to distant organs. Informed by the findings of the KEYNOTE-826 investigation, the U.S. Food and Drug Administration granted approval for pembrolizumab in conjunction with chemotherapy as a primary therapy alternative for recurrent metastatic cervical carcinoma towards the conclusion of 2022 [2]. The present article reviews the local therapies for recurrent cervical carcinoma, like surgical treatment, reprogrammed radiotherapy, and systemic treatments such as systemic chemotherapy, targeted therapy, immunotherapy, etc. Furthermore, the paper examines innovative therapeutic strategies for recurrent metastatic cervical carcinoma, providing an analysis and overview of current treatment modalities. A literature search was performed in PubMed, Scopus, and Web of Science using the keywords ‘recurrent cervical cancer,’ ‘radiotherapy,’ and ‘targeted therapy,’ focusing on studies published between 2015 and 2024.

## 2. Current Treatment Standard for Recurrent Metastatic Cervical Cancer

A significant proportion of individuals diagnosed with locally advanced cervical carcinoma (FIGO stages IB2 to IVA) may benefit from a multimodal strategy that integrates platinum-based concurrent chemoradiation with high-dose-rate intracavitary brachytherapy, leading to the possibility of complete tumor eradication and long-term remission [3,4]. In instances of unresectable pelvic recurrence, the median survival typically ranges from 7 to 12 months across the majority of cases [3,5]. Cisplatin is commonly acknowledged as the fundamental therapeutic ingredient in the treatment of recurrent metastatic cervical carcinoma. A vital clinical challenge, however, is that most patients presenting with recurrence or metastasis have previously undergone cisplatin-based concurrent chemoradiation as front-line treatment. Consequently, they may have developed resistance to subsequent single-agent platinum therapy [6,7]. The NCCN guidelines indicate that the combination of platinum-based agents with paclitaxel has been thoroughly assessed and is recognized as a standard first-line therapy for recurrent or metastatic cervical carcinoma [3,4,5,6]. In 2014, the U.S. FDA granted approval for the anti-angiogenic medication bevacizumab, which focuses on vascular endothelial growth factor (VEGF), to be utilized in first-line therapy of recurrent or metastatic cervical carcinoma. The authorization was conferred in light of the findings from the pivotal Phase III GOG-240 investigation (NCT00803062), which revealed a noteworthy enhancement in both median PFS (8.2 versus 5.9 months) and OS (16.8 versus 13.3 months) [7].

## 3. Local Treatment for Pelvic Recurrence

### 3.1. Surgical Treatment

In individuals with cervical carcinoma experiencing pelvic recurrence following previous radiotherapy, the relatively poor response rate to chemotherapy and the limited treatment options make pelvic exenteration a potential therapeutic option for disease recurrence [8]. Current reports indicate that pelvic exenteration is effective in treating gynecological malignant tumors, achieving a 5-year OS rate ranging from 32.3% to 66% [9]. The 2025 NCCN standards indicate that pelvic exenteration is a viable treatment option for individuals with cervical carcinoma who experience central pelvic recurrence following radiotherapy. At present, experts believe that patients undergoing pelvic exenteration should meet the following criteria: (1) cervical cancer with central pelvic recurrence after radiotherapy and/or pelvic exenteration; (2) a recurrent tumor diameter of less than 5 cm; (3) patients whose general condition and concomitant medical conditions are assessed as able to tolerate surgery [10]. Currently, patients with tumor metastasis outside the pelvis, peritoneal metastasis, invasion of the sciatic nerve or lumbosacral plexus nerves, or lymph node metastasis outside the pelvis are considered absolute contraindications for pelvic exenteration.

### 3.2. Re-Irradiation

Re-irradiation refers to the second round of radiotherapy to treat recurrence at the primary tumor site (more than 90 days after the initial radiotherapy). The appropriate selection of indications for re-irradiation is crucial. The interval from the completion of previous radiotherapy to recurrence is a key reference: patients with an interval of ≥2 years have significantly better outcomes, and the minimum interval should generally not be less than one year, with out-of-field recurrence being an exception. The contraindications for re-irradiation include: (1) the presence of a vaginal fistula; (2) a significant bleeding tendency or severe coagulation dysfunction; (3) the existence of severe comorbid conditions. Radiotherapy for cervical cancer differs from that of other tumors. Specifically, in addition to external beam radiotherapy, brachytherapy can be performed on the local tumor due to the anatomy of the cervix. Brachytherapy involves rapid changes in dose gradients, resulting in lower radiation exposure to surrounding tissues. This facilitates a markedly enhanced transmission of doses to the malignant area, concurrently reducing exposure to adjacent tissues, thus attaining optimal tumor control rates. Therefore, the irradiation dose administered in cervical cancer is often much higher than that of other tumors, which is also an important reason for the high cure rate of cervical cancer. However, radical radiotherapy remains a therapeutic option even in cases of cervical cancer recurrence after radiotherapy.

Xiang et al. [11] conducted an analysis involving 73 individuals diagnosed with cervical carcinoma, all of whom demonstrated vaginal recurrence following radiotherapy and subsequently underwent re-irradiation treatment. Subsequent to external beam radiotherapy, patients underwent brachytherapy to attain dosages ranging from 60 to 72 Gy. The 5-year OS rate reached 40%, but nearly half of the patients developed late complications, with 25% experiencing severe intestinal and bladder toxicity. This study was published earlier, and the relatively poor results may be attributed to the limited technology of external and internal irradiation at that time. Lee et al. [12] utilized image-guided brachytherapy to remedy 44 individuals with vaginal recurrence of cervical cancer. Among them, 13 cases underwent re-irradiation, with the total equivalent dose of late-reaction tissue limited to 70–75 Gy in 2 Gy portions. The findings revealed a 2-year OS rate of 55% within the radiotherapy retreatment cohort, in contrast to an 80% rate observed in the cohort that did not undergo retreatment. In a meticulous observational analysis, Kim et al. [13] scrutinized a sample of 125 individuals afflicted with recurrent cervical carcinoma, each of whom had previously undergone radiotherapy. The median dosage of the recurrent external beam radiotherapy was 54Gy, with a range of 40 to 76Gy. The 5-year rates for local failure-free survival, PFS, and OS were recorded at 47.1%, 33.2%, and 66.5%, respectively. It is significant that the incidence of medication-related late complications of grade 2 or higher was recorded at a mere 9.6%. The study’s findings indicate that, with careful management of radiation dose to healthy tissues, salvage reirradiation is a viable and well-tolerated therapeutic strategy for recurrent cervical cancer. Stereotactic body radiotherapy (SBRT) presents a viable therapeutic option for patients experiencing isolated recurrence or oligometastatic disease from cervical cancer [14]. Murray et al. [15] systematically analyzed 205 patients who underwent retreatment stereotactic ablative radiotherapy after pelvic radiotherapy. The median radiation dose and fractionation schedule were 30 Gy/4.5 fractions (15 Gy/3f–60 Gy/3f), with a 1-year local control (LC) rate spanning 51% to 100%. Nine patients exhibited Grade 3 toxicities, while six patients experienced Grade 4 adverse events. Consequently, stereotactic ablation radiotherapy presents a viable treatment strategy for addressing recurrent disease subsequent to initial radiotherapy. Previous pelvic surgical interventions, the administration of anti-angiogenic medications, or a high total radiation dose may elevate the risk of toxicity following stereotactic ablative radiotherapy and subsequent re-irradiation [16]. In addition, several investigations have utilized radioactive particle implantation as a therapeutic approach for individuals suffering from recurrent cervical carcinoma. Brabham et al. [17] reported that Au198 particles were implanted to treat 19 recurrent gynecologic malignancies at a median dosages of 50 Gy. Complete remission was attained in 95% of the subjects, with more than half achieving a state of tumor-free survival, and merely one individual encountered grade 3 toxicity. Li et al. [18] conducted a retrospective study that included 50 patients who underwent re-irradiation. All patients received either volumetric modulated arc therapy (VMAT) alone or VMAT combined with three-dimensional image-guided brachytherapy (3D-IGBT). The median interval from the initial radiotherapy to re-irradiation was 12 months. With a median follow-up time of 22 months (range: 4–59 months), the median progression-free survival (PFS) was 14 months and the median overall survival (OS) was 26 months. An interval of more than 12 months between the two courses of radiotherapy was significantly associated with improved local control (LC) and PFS (*p* ≤ 0.05), but not with OS benefit (*p* > 0.05). Hye Jin Kang et al. [19] retrospectively analyzed 22 patients with recurrent cervical cancer who received intensity-modulated radiotherapy (IMRT) for pelvic recurrence. With a median follow-up period of 15 months (range: 3–120), the overall response rate was 63.6%. The 1-year and 2-year overall survival (OS) rates were 68.2% and 25.0%, respectively. For patients with recurrent cervical cancer who had previously undergone radiotherapy, re-irradiation using IMRT proved to be a safe and effective treatment strategy. The interval between radiation courses, tumor size, response to re-irradiation, and radiation dose were identified as key factors influencing both efficacy and safety.

## 4. Systemic Therapy for Pelvic Recurrence

### 4.1. Targeted Therapy

**Figure 1:**
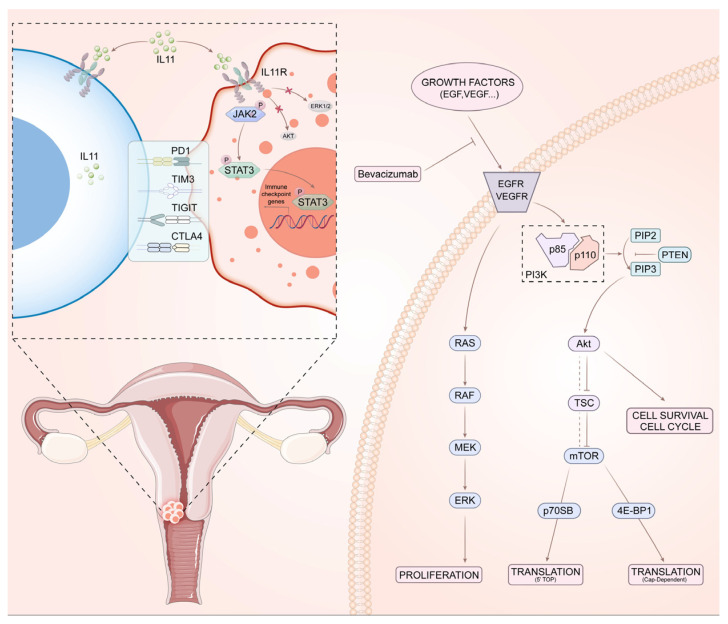
Flowchart of the Mechanism of Action of Targeted Drugs. VEGF/VEGFR Pathway: Vascular Endothelial Growth Factor (VEGF) binding to its receptor (VEGFR) promotes angiogenesis. Bevacizumab, a monoclonal antibody, targets VEGF to inhibit this process. EGFR Pathway: Ligand binding to the Epidermal Growth Factor Receptor (EGFR) activates downstream signals (e.g., RAS-RAF-MEK-ERK) that drive cell proliferation. TKIs and monoclonal antibodies (e.g., Cetuximab) target this axis. PI3K/AKT/mTOR Pathway: Activation of Phosphatidylinositol 3-Kinase (PI3K) leads to Akt and mTOR activation, regulating cell survival and growth. This pathway is negatively regulated by PTEN. Abbreviations: IL-11: Interleukin-11; PD-1: Programmed Cell Death Protein 1; TIM-3: T-cell Immunoglobulin and Mucin domain-containing protein 3; TIGIT: T cell immunoreceptor with Ig and ITIM domains; CTLA-4: Cytotoxic T-Lymphocyte-Associated protein 4; EGF: Epidermal Growth Factor; EGFR: Epidermal Growth Factor Receptor; VEGFR: Vascular Endothelial Growth Factor Receptor; ERK: Extracellular Signal-Regulated Kinases; MEK: MAPK/ERK Kinase; RAF: Rapidly Accelerated Fibrosarcoma; RAS: Rat Sarcoma; p70SB: p70 Ribosomal Protein S6 Kinase; 4E-BP1: eIF4E-Binding Protein 1; mTOR: Mechanistic Target of Rapamycin; TSC: Tuberous Sclerosis Complex; Akt: Ak strain transforming; PTEN: Phosphatase and Tensin Homolog (deleted on chromosome 10); PIP2: Phosphatidylinositol 4,5-bisphosphate; PIP3: Phosphatidylinositol (3,4,5)-trisphosphate; PI3k: Phosphatidylinositol 3-Kinase; p85: p85 regulatory subunit of PI3K; p110: p110 catalytic subunit of PI3K.The figure is original using Adobe illustrator under an appropriate academic license.

Targeted therapy refers to a treatment strategy designed to selectively inhibit molecular targets—such as genes or proteins—that play critical roles in cancer development. (Figure 1). The contemporary approaches to targeted treatment for cervical carcinoma primarily encompass anti-angiogenic agents, poly (ADP-ribose) polymerase (PARP) and small-molecule tyrosine kinase inhibitors. Each of these therapeutic classes has demonstrated considerable effectiveness in the management of recurrent metastatic cervical carcinoma (Table 1). VEGF inhibitors: adding anti-angiogenic drugs to conventional chemotherapy.

Angiogenesis represents a fundamental physiological mechanism vital for meeting the oxygen demands of cells, tissues, and organs. This process is a crucial mechanism in cancer progression, radiotherapy resistance, and metastasis. The therapeutic targeting of excessive angiogenesis represents a significant strategy in managing autoimmune, inflammatory, and malignant diseases [31]. VEGF, a versatile pro-angiogenic cytokine, serves as a crucial intermediary of angiogenesis in cervical carcinoma [32]. The process of tumor angiogenesis plays a pivotal role in the progression of cervical carcinoma, leading to anti-angiogenic medication emerging as a significant area of clinical investigation [33].

Bevacizumab is a monoclonal antibody that functions by focusing on VEGF, consequently obstructing the formation of new blood vessels within tumors. The amalgamation of bevacizumab with chemotherapy facilitates the normalization of blood vessels and improves vascular permeability. This phenomenon enhances the penetration of immune cells within the tumor and elevates the tissue’s responsiveness to chemotherapeutic agents [34]. In light of the findings from the GOG 240 trial, the US FDA conferred approval for bevacizumab as a treatment for metastatic, persistent, or recurrent cervical carcinoma on 14 August 2014. This RCT, involving around 450 individuals diagnosed with severe cervical carcinoma, culminated in the authorization of the first novel therapeutic agent for cervical carcinoma in eight years. An appreciable enhancement was noted within the cohort receiving combination therapy; the response rate elevated to 48% from 36%, while the median PFS was prolonged to 8.2 months, in contrast to 5.9 months seen in the chemotherapy-only cohort [4,10]. However, patients with cervical carcinoma who have received prior radiotherapy and are subsequently given bevacizumab face a heightened risk of complications such as urinary tract or rectovaginal fistulas, deep vein thrombosis, bleeding, and intestinal perforation [3]. PARP constitutes a group of enzymes that are integral to various biological functions, encompassing gene transcription, DNA damage reconstruction, and apoptosis. PARP inhibitors operate by obstructing the repair mechanisms of DNA single-strand breaks (SSBs), resulting in the buildup of breaks in the double-strand. In cancer cells lacking the capability for homology repair, this leads to severe and irreparable DNA damage [35]. A promising avenue in targeted cancer therapy involves the application of PARP inhibitors in the management of BRCA-mutant malignancies, particularly breast and ovarian malignancies [36,37]. PARP inhibitors are currently under investigation for cervical cancer, with research ongoing in the preclinical setting. Similar to non-small cell lung cancer, mesothelioma, and ovarian cancer cell lines, Michels et al. [38] established a cisplatin-resistant cervical cancer (HeLa) cell line. These cell lines exhibit high levels of PAR and PARP1, with constitutive hyperactivation of PARP1. The research group also observed that PAR levels identified in tumor cells and xenografts overexpressing PARP1 more accurately predicted responses to PARP inhibitors than PARP1 expression alone. The results suggest that poly(ADP-ribose) (PAR) could serve as a more reliable predictive biomarker for evaluating therapeutic outcomes for suppression of PARP in cervical carcinoma. Trials conducted both in the laboratory and in real life on a selection of primary cervical cancer cell lines revealed that the PARP inhibitor olaparib markedly inhibited PARP activity [39,40,41]. A case report illustrated total tumor remission in a cervical carcinoma patient subsequent to a combination therapy involving olaparib and bevacizumab, following the administration of radiotherapy and chemotherapy [29]. A Phase I clinical investigation is presently enlisting participants diagnosed with cervical carcinoma and various gynecological carcinomas to assess the effectiveness of olaparib in conjunction with carboplatin for the management of refractory or recurrent disease (NCT01237067).

A separate Phase I/II clinical investigation examined the efficacy of combining veliparib with cisplatin and paclitaxel in cases of severe, persistent, or recurrent cervical carcinoma. A total of 34 individuals, who were deemed ineligible for curative therapy, were enrolled in the study. In the cohort of 29 evaluable patients, the findings revealed an objective RR of 60%, with a median PFS of 6.2 months and a median OS of 14.5 months. Future preclinical and clinical research is anticipated to further elucidate the curative value of PARP inhibitors in the context of cervical carcinoma [42].

A Phase II clinical investigation was undertaken to assess the effectiveness of bevacizumab in conjunction with rucaparib, involving 33 participants diagnosed with recurrent or endometrial carcinoma. Among the 28 patients assessed for response, an objective RR was noted in 17% of individuals with endometrial carcinoma and 14% of those with cervical carcinoma. The median duration of PFS was recorded at 3.8 months, while the median OS was noted to be 10.1 months [30]. The FDA has granted approval for the PARP inhibitor rucaparib to be utilized as maintenance treatment in cases of recurrent cervical cancer [43].

According to one study, cervical cancer cell nuclei exhibited approximately a two-fold increase in PARP activity compared to normal cervical cells [44]. Consequently, PARP represents a promising therapeutic target for individuals experiencing recurrent metastatic cervical carcinoma. Given the insufficient robust efficacy data for recurrent and metastatic cervical carcinoma, clinical trials are presently assessing the therapeutic potentials of veliparib, rucaparib, and niraparib in this context.

#### 4.1.1. Tyrosine Kinase Inhibitors

Tyrosine kinases represent a category of enzymes that facilitate the phosphorylation process by transferring phosphate groups from ATP to tyrosine residues on designated protein targets, a mechanism essential for maintaining normal cellular homeostasis [45]. Ligands like VEGF and EGF bind to the extracellular domains of receptor tyrosine kinases (RTKs), triggering their activation and facilitating dimerization. The aberrant signaling pathways characteristic of cancer cells facilitate uncontrolled growth and contribute to the process of oncogenic transformation [46]. Numerous small-molecule inhibitors impede intracellular communication through their interaction with the ATP-binding catalytic site located within the tyrosine kinase domain of VEGFR [47].

Nimo represents a groundbreaking advancement in the realm of TKIs, specifically designed to specifically inhibit VEGFR2, the primary receptor for VEGF-A, which plays a pivotal role in the process of angiogenesis [20]. A systematic review conducted by Huang et al. [48] involving seven Phase II clinical trials and retrospective investigations with a total of 243 patients revealed a combined median PFS of 5.19 months and a median OS of 10.63 months. The findings demonstrate that apatinib shows promising effectiveness and an acceptable safety rating in addressing chronic or recurrent cervical carcinoma.

Pazopanib is an intravenous multi-kinase inhibitor that targets VEGFR-1, VEGFR-2, VEGFR-3, PDGFR-α, PDGFR-β, and c-Kit. In contrast, lapatinib functions as a dual tyrosine kinase inhibitor, specifically addressing the EGFR and human epidermal growth factor receptor 2 [21]. A Phase II RCT encompassing 230 individuals diagnosed with Stage IVB persistent or recurrent cervical carcinoma assessed the effectiveness of pazopanib monotherapy, lapatinib monotherapy, and their combination therapy. In the monotherapy cohort, 152 patients were allocated at random to receive either pazopanib (*n* = 74) or lapatinib (*n* = 78). 62% of these individuals had encountered a recurrence of their tumors. The median OS was recorded at 50.7 weeks for pazopanib, in contrast to 39.1 weeks for lapatinib, with corresponding response rates of 9% and 5% [49]. While effective to a degree, both therapeutic regimens exhibit diminished effectiveness in cases of severe and recurrent cervical carcinoma [50]. At present, lapatinib and pazopanib do not qualify as standard therapeutic modalities for cervical carcinoma, necessitating additional investigation.

Cediranib functions as an inhibitor of PDGFR-α, VEGFR1–3 tyrosine kinase, and the stem cell factor receptor, thereby blocking VEGF-mediated signaling pathways [22]. A random-access, double-blind, placebo-controlled Phase II investigation was carried out involving 69 individuals diagnosed with recurrent or metastatic cervical carcinoma. The findings indicated that the incorporation of cediranib into the normal chemotherapy regimen markedly prolonged the median PFS to 8.1 months, compared to 6.7 months observed in the placebo cohort [51].

Nintedanib is a powerful oral inhibitor that specifically targets crucial RTKs associated with angiogenesis, namely VEGFR 1–3, FGFR 1–3, and PDGFR-α/β [23]. In a double-blind Phase II randomized trial involving individuals with newly discovered chronic or first-line recurrent cervical carcinoma, Vergote et al. [52] illustrated that the incorporation of nintedanib alongside paclitaxel and carboplatin yielded a median OS of 21.7 months, in contrast to 16.4 months observed in the control group. These results underscore the promising potential of nintedanib in this treatment setting.

Macka et al. [24] undertook a single-arm, multicenter Phase II investigation involving individuals with locally advanced or metastatic cervical carcinoma to assess the efficacy of sunitinib, an orally taken TKI that specifically targets VEGFR1–3, PDGFR-α/β, c-Kit, and FLT3 receptors. Of the 16 participants, 84% attained stable disease, with a median PFS of 3.5 months (95% CI: 2.6–7.0). The present amount of proof regarding the effectiveness and safety profile of sunitinib monotherapy for cervical carcinoma remains inconclusive, highlighting the imperative for further investigation.

#### 4.1.2. Antibody-Drug Conjugates

In recent years, antibody-drug conjugates (ADCs) have emerged as a novel class of therapeutics, garnering considerable interest for their potential in recurrent or metastatic cervical cancer. By enabling targeted delivery of cytotoxic agents, ADCs represent a promising strategy in precision oncology. Preclinical and clinical research on ADCs for the treatment of cervical cancer is being actively conducted worldwide. These ADCs offer additional therapeutic options for patients with advanced cervical cancer, particularly for those who have progressed after multiple lines of therapy, and hold the potential to improve clinical outcomes. Tumor-associated antigens (TAAs) with potential for ADC targeting in cervical cancer include TF, human epidermal growth factor receptor 2 (HER2), trophoblast cell surface antigen 2 (Trop-2), mesothelin, and Nectin cell adhesion molecule 4 (Nectin-4) (Table 2).

##### Tissue Factor (TF)

Tisotumab vedotin (TV), the first ADC approved for the treatment of cervical cancer, consists of a human TF-specific mAb, a protease-cleavable linker, and the highly potent cytotoxic payload monomethyl auristatin E (MMAE)—a microtubule disrupting agent [60]. The innovaTV 204/GOG-3023/ENGOT-cx6 trial, a multicenter, single-arm Phase II study, evaluated the efficiency and safety of tisotumab vedotin in a cohort of 102 individuals diagnosed with recurrent or metastatic cervical carcinoma. The findings revealed an objective RR of 24%, which includes a CRR of 7% and a PR of 17%. These findings indicate that tisotumab vedotin exhibits clinically significant and sustained antitumor efficacy in patients experiencing recurrent or metastatic cervical carcinoma [25]. The Phase III innovaTV 301 trial was launched to evaluate the effectiveness of tisotumab vedotin in the second- or third-line treatment context, building upon previous research outcomes. The research involved 502 participants diagnosed with recurrent or metastatic cervical carcinoma, who were randomized to be given either tisotumab vedotin or the investigator’s selected single-agent chemotherapy. The findings revealed a statistically significant enhancement in OS for the tisotumab vedotin cohort, with a median OS of 11.5 months in contrast to 9.5 months observed in the chemotherapy cohort [61].

##### Human Epidermal Growth Factor Receptor 2 (HER2)

Fam-trastuzumab deruxtecan-nxki (T-DXd) is an antibody-drug conjugate (ADC) composed of trastuzumab, a humanized anti-HER2 monoclonal antibody, conjugated to a topoisomerase I inhibitor payload (DXd) via a cleavable tetrapeptide-based linker [53]. T-DXd has been approved for the treatment of metastatic HER2-positive or HER2-low breast cancer (BC), HER2-mutant non-small cell lung cancer (NSCLC), metastatic HER2-positive gastric/gastroesophageal junction cancer (GC/GEJC).

IBI354 is a novel antibody-drug conjugate (ADC) that delivers a topoisomerase I inhibitor payload via the trastuzumab antibody. Preliminary results from a Phase I study (NCT05636215) in advanced gynecologic cancers demonstrated promising efficacy in a cohort of 14 patients with HER2 2+/3+ cervical or endometrial cancer, showing an objective response rate (ORR) of 57.1% [54].

##### Trophoblast Cell Surface Antigen 2 (Trop-2)

Sacituzumab govitecan (SG) is a first-in-class, FDA-approved anti-Trop-2 antibody-drug conjugate (ADC). It is composed of the hRS7 antibody conjugated to SN-38—an active metabolite of irinotecan and a potent topoisomerase I inhibitor—via a hydrolyzable CL2A linker [55]. The feasibility of SG for treating recurrent or metastatic cervical cancer has also garnered significant attention.

EVER-132-003 (NCT05119907) is a multicenter, single-arm, open-label phase II study evaluating SG in patients with solid tumors. Cohort C of this study enrolled 18 Chinese adult patients with recurrent or metastatic cervical cancer who had disease progression after receiving ≥1L of systemic therapy. Interim analysis data demonstrated encouraging antitumor activity of SG in recurrent or metastatic cervical cancer patients, with an ORR of 50% and a median DoR of 9.2 months. The DCR was 94% and the median PFS in this cohort was 8.1 months [56].

An ongoing phase II basket trial (NCT05642780) is assessing the efficacy and safety of SKB-264 combined with pembrolizumab in patients with recurrent or metastatic cervical cancer. The study enrolled individuals who had experienced disease progression during or after platinum-based doublet chemotherapy and had received no more than two prior lines of systemic therapy for recurrent or metastatic disease. Preliminary results demonstrate an objective response rate (ORR) of 57.9%, with the median duration of response (DoR) not yet reached [54].

##### Mesothelin

RC88 is a novel antibody-drug conjugate (ADC) composed of a humanized anti-mesothelin monoclonal antibody conjugated to the cytotoxic agent monomethyl auristatin E (MMAE) via a cleavable linker. A single-arm, open-label, phase I/II trial (NCT04175847) is evaluating RC88 in patients with mesothelin-expressing advanced solid tumors. As of 19 December 2023, 164 patients who had progressed on standard therapies were enrolled, including a cohort of 18 with cervical cancer. Among 17 evaluable cervical cancer patients, the majority (64.7%) had received ≥2 prior lines of therapy, and 70.5% had prior exposure to both platinum-doublet chemotherapy and a PD-(L)1 inhibitor. In this heavily pretreated cohort, the objective response rate (ORR) was 35.3% [57].

##### Nectin Cell Adhesion Molecule 4 (Nectin-4)

9MW2821 is a novel Nectin-4-targeting antibody-drug conjugate (ADC) independently developed by Mavis Biologics. It is composed of a site-specifically conjugated humanized monoclonal antibody, an enzymatically cleavable valine-citrulline linker, and the cytotoxic payload monomethyl auristatin E (MMAE) [58]. A first-in-human, open label, multicenter phase I/II study (NCT05216965) evaluating the safety and preliminary efficacy of 9MW2821 enrolled 274 patients with Nectin-4-positive solid tumors who failed ≥1L of systemic therapy. In 53 evaluable recurrent or metastatic cervical cancer patients treated with 9MW2821, the ORR was 32.1%, and the DCR reached 81.1%. 9MW2821 is the first Nectin-4-targeted ADC to demonstrate antitumor activity in patients with cervical cancer [59].

#### 4.1.3. Epidermal Growth Factor Receptor-Targeted Therapy

EGFR is a transmembrane protein that is crucial in various cell signaling networks, fundamental to the regulation of cell proliferation, differentiation, division, and survival [62]. The expression of EGFR is elevated in cervical carcinoma cells [63,64]. After binding with its ligand, EGFR forms dimers and activates downstream signal transduction pathways, thereby inhibiting apoptosis and promoting cell formation and angiogenesis. Blocking EGFR signal transduction can increase tumor sensitivity to radiotherapy and chemotherapy, emphasizing the significance of EGFR as a target gene in the therapeutic approach to cervical carcinoma [65].

Cetuximab is a monoclonal antibody targeting EGFR, demonstrating the ability to inhibit tumor proliferation in cervical tumor cell lines [66]. It can be administered securely alongside entire doses of carboplatin and paclitaxel [67]. A randomized, prospective, open-label, multicenter Phase II clinical investigation (NCT00997009) involved the enrollment of 108 individuals who were randomly assigned to receive either a regimen of paclitaxel plus carboplatin (CP) or a regimen combining paclitaxel, carboplatin, and cetuximab (CP-CET). The research ultimately revealed that incorporating cetuximab into the regimen of carboplatin and paclitaxel failed to produce a statistically significant enhancement in either PFS or OS among individuals suffering from terminal or recurrent cervical carcinoma. Therefore, further inquiry is essential to conclusively determine the therapeutic efficiency of cetuximab in the management of recurrent metastatic cervical carcinoma.

Gefitinib, a first-generation TKI that specifically targets the EGFR, is widely employed in the treatment of lung cancer [62,67]. By obstructing the activity of EGFR tyrosine kinase, it effectively halts the stimulation of subsequent signaling channels. This disruption impedes the advancement of the cell cycle, curtails epithelial–mesenchymal differentiation, and triggers apoptosis in cancerous cervical cells, thereby culminating in a suppression of tumor cell proliferation and development [68]. A study conducted by Sharma et al. [69] examined individuals with severe cervical carcinoma who had experienced progression following first-line chemotherapy. These individuals were administered gefitinib, leading to a median PFS of 4 months and a median OS of 5 months. The researchers concluded that gefitinib exhibited restricted effectiveness as a standalone treatment within that cohort. However, they proposed that its integration with other therapeutic agents could yield more favorable outcomes. A Phase II investigation led by Goncalves et al. [26] evaluated the effectiveness of gefitinib in severe cervical carcinoma, revealing that 20% of the participants experienced stable disease (SD) with a median duration of 111.5 days. Compared with traditional chemotherapy, gefitinib is relatively affordable. Additionally, its oral formulation provides convenience for patients with limited access to healthcare facilities compared to intravenous treatment. While gefitinib demonstrates commendable effectiveness and safety, it remains imperative to pursue additional investigations to refine its application in the management of severe cervical carcinoma.

### 4.2. Immunotherapy

Immune cells express immune checkpoint molecules on their surfaces, which are involved in regulating immune responses and preventing autoimmunity during infection [70]. Most cervical cancers are caused by HPV infection, which weakens the immune system [71]. Immune checkpoint inhibitors block immunosuppressive signals, allowing immune cells to specifically target tumor cells (Table 3). Notably, programmed death protein 1 and its ligands (PD-L1, PD-L2) and CTLA-4 are the most commonly used immune checkpoint inhibitors [44].

#### 4.2.1. PD-1 Inhibitors

The preponderance of cervical carcinoma instances is linked to infection by oncogenic types of human papillomavirus (HPV), that may undermine the patient’s defences. Consequently, immunotherapy, particularly application of immune checkpoint inhibitors has surfaced as an exceptionally beneficial medicinal strategy [71]. PD-1 serves as an immune checkpoint receptor, present on the surface of T cells, and plays a crucial role in the regulation of immune responses [27]. Tumor cells exploit immune evasion through the expression of programmed cell PD-L1, a ligand that binds to the PD-1 receptor on immune cells; this interaction collectively suppresses T-cell activity and facilitates escape from immune surveillance [80]. The interplay between PD-1 and its ligand is fundamental in sustaining immune tolerance, as it suppresses stimulation of T cells and fosters adaptive immune resistance [81]. Consequently, the blockade of PD-1 or PD-L1 has the potential to restore T-cell vigour, enhance T-cell efficacy, modify the tumor immune microenvironment, and strengthen the patient’s antitumor immune response [44].

##### Pembrolizumab

The efficiency of targeted PD-1 or PD-L1 inhibition in the management of cervical carcinoma has been firmly established through multiple clinical trials [28]. Pembrolizumab, the first anti-PD-1 agent to be developed, was evaluated in the KEYNOTE-028 and KEYNOTE-158 clinical trials [82]. The incorporation of the anti-PD-1 monoclonal antibody pembrolizumab into chemotherapy, whether combined with bevacizumab or not, demonstrated notable enhancements in OS and PFS for patients suffering from recurrent or metastatic cervical cancer, in contrast to the regimen involving placebo and chemotherapy. Importantly, the observed adverse events were deemed manageable [83]. Their findings revealed that a notably greater percentage of individuals in the pembrolizumab cohort indicated enhancements in General Health Status Quality of Life (GHS-QoL) relative to the placebo cohort (42% vs. 29%), thus substantiating the therapeutic advantages of pembrolizumab in cases of metastatic or recurrent cervical carcinoma. The overall survival outcomes of pembrolizumab versus placebo in combination with chemoradiotherapy were reported in the ENGOT-cx11/GOG-3047/KEYNOTE-A18 trial, a randomized, double-blind, Phase III study. This trial enrolled 1060 treatment-naïve patients with high-risk, locally advanced cervical cancer, randomizing 529 to the pembrolizumab–chemoradiotherapy group and 531 to the placebo–chemoradiotherapy group. After a median follow-up of 29.9 months, the median overall survival had not been reached in either group. The 36-month overall survival rate was 82.6% for patients receiving pembrolizumab plus chemoradiotherapy, compared to 74.8% for those in the placebo plus chemoradiotherapy group [83].

The Phase III, double-blind KEYNOTE-826 trial evaluated patients with persistent, recurrent, or metastatic cervical cancer who were treated with a combination of platinum-based chemotherapy (with or without bevacizumab) plus either pembrolizumab or a placebo. The median follow-up was 39.1 months. The results indicated that median OS was significantly longer in the pembrolizumab plus chemotherapy group compared to the placebo plus chemotherapy group across all PD-L1 expression subgroups. Within the PD-L1 CPS ≥1 population (*n* = 548), median overall survival (OS) was significantly longer in the pembrolizumab–chemotherapy group at 28.6 months, compared to 16.5 months in the placebo–chemotherapy group. Among all patients (*n* = 617), a median OS of 26.4 months was achieved in the pembrolizumab plus chemotherapy group, which was significantly longer than the 16.8 months of the placebo plus chemotherapy group. Similarly, in the cohort with a combined positive score (CPS) of 10 or higher (*n* = 317), the median overall survival was significantly prolonged in the pembrolizumab–chemotherapy group compared to the placebo–chemotherapy group (29.6 months vs. 17.4 months). These findings indicate that the addition of pembrolizumab to chemotherapy, with or without bevacizumab, offers clinically meaningful overall survival benefits for patients with persistent, recurrent, or metastatic cervical cancer [2]. A key limitation of the KEYNOTE-826 trial lies in the maintenance of its double-blind design. Although the trial itself was double-blinded, the characteristic immune-related adverse events (irAEs) associated with immune checkpoint inhibitors such as pembrolizumab may have allowed investigators and patients to deduce treatment allocation. This could potentially introduce performance bias in the assessment of certain subjective endpoints or in the management of adverse events. Furthermore, while the trial’s allowance for bevacizumab use in combination with chemotherapy, or not, reflects real-world clinical practice, any imbalance in the use of bevacizumab between the treatment groups could have exerted a confounding influence on the final overall survival (OS) results.

The current first-line standard of care for recurrent or metastatic cervical cancer consists of bevacizumab combined with chemotherapy. Supported by findings from the KEYNOTE-028, KEYNOTE-158, and KEYNOTE-826 clinical trials, the therapeutic strategy has been enhanced by incorporating the programmed death protein-1 inhibitor pembrolizumab alongside platinum-based chemotherapy into this established regimen [84]. Use with or without bevacizumab was found to significantly improve PFS and OS in PD-L1-positive patients [85]. Based on the results of KEYNOTE-826, pembrolizumab in combination with platinum/paclitaxel and bevacizumab was approved as a first-line treatment regimen for recurrent/metastatic cervical cancer with significant PD-L1 expression [86].

##### Enlonstobart Injection

SG001 (Enlonstobart Injection) is a fully humanized, high-affinity immunoglobulin G4 monoclonal antibody directed against PD-1, which it inhibits by blocking its binding to the ligands PD-L1 and PD-L2. In a Phase IB clinical trial (NCT03852823), SG001 demonstrated promising efficacy and an acceptable safety profile as a monotherapy in patients with recurrent or metastatic cervical cancer. A total of 91 patients were enrolled, with 98.9% of patients having previously received platinum-based chemotherapy. Specifically, 91.2% had previously received radiotherapy, 80.2% had squamous cell carcinoma, 85.7% had distant metastasis, and 47.3% were PD-L1-positive (CPS ≥ 1). The median progression-free survival (PFS) was 5.5 months, with a 6-month PFS rate of 43.8%. As of the data cutoff date, 72.5% of patients were still alive, resulting in a 12-month overall survival (OS) rate of 65.8%. Confirmed responses were also observed in PD-L1-negative patients (*n* = 45), with a median PFS of 4.3 months. Compared with previous studies, this study showed that the ORR of SG001 (Enlonstobart Injection) monotherapy was higher than that of other second-line treatments in both PD-L1-positive and PD-L1-negative populations [84].

A Phase II study evaluated the efficacy of SG001 (Enlonstobart Injection) in patients with PD-L1-positive recurrent/metastatic cervical cancer, enrolling a total of 107 patients with a median follow-up of 14.0 months. The overall response rate (ORR) was 29.0%, including 2 cases of complete response and 29 cases of partial response. Notably, 52.3% of patients experienced tumor shrinkage, with 34.6% achieving ≥30% tumor shrinkage. Patients with PD-L1 CPS ≥ 5 exhibited a higher ORR than those with PD-L1 CPS 1 ≤ CPS < 10 and PD-L1 CPS 10 ≤ CPS < 50. In addition, patients aged ≥ 65 years demonstrated a higher ORR than those aged < 65 years. The median PFS was 3.1 months, with estimated PFS rates of 41.3% at 6 months, 31.3% at 9 months, and 29.9% at 12 months. The median OS has not been reached, with estimated OS rates of 68.4% at 12 months and 54.6% at 18 months [87].

##### Atezolizumab

The BEATcc study (ENGOTCx10/GEICO 68-C/JGOG1084/GOG-3030, NCT03556839) is a multicenter, randomized Phase III trial evaluating the efficacy of atezolizumab (anti-PD-L1 antibody) in combination with platinum-based chemotherapy plus bevacizumab in patients with metastatic, recurrent, or persistent cervical cancer (regardless of PD-L1 expression levels) [85]. The results showed a 38% reduction in the risk of progression or death in the atezolizumab group (median PFS of 13.7 months and 10.4 months, respectively), with a significant improvement in median OS (32.1 months vs. 22.8 months). As opposed to the KEYNOTE-826 study, this study did not stratify patients based on PD-L1 CPS but conducted an exploratory analysis of results based on PD-L1 status. Currently, atezolizumab is approved for the treatment of bladder cancer, lung cancer, and liver cancer, but has not yet been approved by the FDA for use in cervical cancer patients. A key design choice of the BEATcc trial was that it did not stratify patients based on PD-L1 expression levels. Although a post hoc analysis suggested that the benefit of atezolizumab appeared to be independent of PD-L1 status, the lack of pre-specified stratification could have led to an uneven distribution of baseline PD-L1 expression between the groups, thereby introducing selection bias. Furthermore, the open-label design of this trial is less objective than a double-blind design, potentially introducing assessment bias, particularly in the evaluation of progression-free survival (PFS) which was assessed by the investigators.

##### Cemiplimab

The Phase III, multicenter, open-label trial EMPOWER Cervical 1/GOG-3016/ENGOT-cx9 evaluated the efficacy of the anti-PD-1 antibody cemiplimab in patients with cervical cancer who progressed after first-line platinum-based chemotherapy [88]. A total of 608 patients were enrolled and randomly assigned to receive cemiplimab or investigator-selected single-agent chemotherapy, regardless of PD-L1 status. The median follow-up duration was 47.3 months. The median overall survival (OS) for patients receiving cemiplimab and other single-agent chemotherapy was 11.7 months and 8.5 months, respectively. OS benefits were observed in both PD-L1-positive and PD-L1-negative populations [88], although a larger proportion of PD-L1-negative patients showed poorer performance status in the cemiplimab group compared to the chemotherapy group (61.4% and 47.9%) [77]. A validated survey revealed that patients treated with cemiplimab had improved quality of life and physical function compared to patients receiving chemotherapy. Cemiplimab was initially approved for the treatment of skin and lung cancer but has not yet been approved by the FDA for the treatment of cervical cancer.

##### Nivolumab

Nivolumab is a programmed death receptor-1 (PD-1) inhibitor that offers a new treatment option for patients with advanced disease [78]. Rodrigues et al. [74] conducted the Phase I, open-label, multicenter NiCOL trial (NCT03298893) to determine the safety, tolerability, and immune-related adverse events of nivolumab in combination with concurrent chemoradiotherapy in patients with locally advanced disease. A total of 16 patients with locally advanced cervical cancer were enrolled, with a median follow-up of 23.8 months, ORR of 93.8%, CRR of 50%, and 2-year PFS of 75%, thereby confirming the efficacy of nivolumab combined with radical chemoradiotherapy. Santin et al. [89] conducted a Phase II trial (Nct02257528/Nrg-Gy002) to evaluate the efficacy of nivolumab in the treatment of persistent or recurrent cervical cancer. A total of 25 patients were included, with a median age of 45 years. PD-L1 expression (≥ 1%) was detected in 77.3% of the samples. One case of partial response (4%) was observed, with a response duration of 3.8 months. The 6-month PFS and OS rates were 16% and 78.4%, respectively. Considering the relatively small number of enrolled patients and short follow-up duration, nivolumab demonstrated low antitumor activity. Petre et al. [90] conducted a systematic review of the efficacy of nivolumab in the treatment of advanced or metastatic cervical cancer, comprising a total of 80 patients, with a mean age of 48 years. Among the included patients, 71% were PD-L1-positive. The median progression-free survival (PFS) at 6 months was 50%, highlighting the significant potential of nivolumab in managing advanced disease stages. These findings also highlight the impact of PD-L1 status on response rates. Further clinical studies are required to confirm the benefits of nivolumab for patients with advanced and metastatic cervical cancer.

##### Zimberelimab

Zimberelimab is an anti-PD-1 monoclonal antibody. A Phase II, single-arm, open-label clinical study enrolled 105 patients with PD-L1-positive recurrent or metastatic cervical cancer who had progressed after first-line or subsequent first-line chemotherapy. The median age was 51 years, with a median follow-up duration of 16.9 months. The objective response rate was 27.6%, and the disease control rate was 55.2%. In this study, the median duration of response was not reached. The median overall survival was 16.8 months, and the median progression-free survival was 3.7 months. Zimberelimab is currently approved in China for the treatment of recurrent or metastatic cervical cancer and recurrent or refractory classical Hodgkin lymphoma [75,79].

#### 4.2.2. PD-L1 Inhibitors

Socazolimab represents an innovative and greatly targeted totally human recombinant anti-PD-L1 monoclonal antibody, exhibiting two systems that work in concert to enhance antitumor efficacy. An et al. [91] conducted an open, Phase I dose-escalation clinical trial to investigate the effectiveness and safety of socazolimab. A total of 104 individuals participated in the investigation. Specifically, 12 individuals participated in the dose-escalation stage, resulting in 1 complete remission and 2 incomplete remissions. An aggregate of 92 individuals advanced to the dose-expansion stage, with 59.3% exhibiting a PD-L1 CPS of ≥1. This cohort achieved an overall response rate of 15.4%, alongside a median progression-free survival of 4.44 months and an mOS of 14.72 months. In addition, 16.7% of individuals who tested positive for PD-L1 exhibited an ORR of 16.7%, while 16.7% of those who tested negative for PD-L1 also demonstrated an ORR of 16.7%. The ORR for PD-L1-negative individuals was 17.9%. The findings of the trial indicate that socazolimab exhibits a noteworthy effectiveness and safety record in addressing recurrent or metastatic cervical carcinoma.

Xu et al. [92] documented an incident involving a Chinese individual diagnosed with severe cervical carcinoma accompanied by multiple metastases, who exhibited notable tumour regression following therapy with socazolimab. Currently, the patient is still undergoing immunotherapy, showing sustained remission.

#### 4.2.3. CTLA-4 Inhibitors

Ipilimumab functions as an antibody targeting CTLA-4. A prospective Phase I clinical trial has engaged 21 individuals diagnosed with cervical carcinoma at stages IB2 to IVA, all of whom presented with positive pelvic lymph nodes, para-aortic lymph nodes, or a combination of the two. Of the participants, 18 patients (86%) successfully underwent four cycles of ipilimumab, while 3 patients (14%) accomplished two cycles of the treatment. The 12-month OOS rate was 90%, and the PFS rate was 81%. Following concurrent chemoradiotherapy, the quantity of PD-1-expressing T cells increased, and sequential ipilimumab was administered to maintain this level [93].

#### 4.2.4. Dual Checkpoint Inhibitors

Certain clinical trials are exploring the efficacy of immunotherapy and various treatment amalgamations as second-line or subsequent therapies for recurrent or metastatic cervical carcinoma [94].

Cadonilimab is a bispecific monoclonal antibody that targets both PD-1 and cytotoxic T lymphocyte-associated antigen-4 (CTLA-4). The Phase II COMPASSION-13 trial assessed the effectiveness of cadonilimab when used in conjunction with first-line cisplatin or carboplatin and paclitaxel, with or without the addition of bevacizumab, involving a cohort of 45 patients diagnosed with recurrent or metastatic cervical carcinoma [79]. In the cohort not administered bevacizumab, the ORR for the cadonilimab 10 mg/kg and 15 mg/kg cohorts were recorded at 66.7% and 68.8%, respectively. In contrast, the ORR for the cadonilimab 10 mg/kg in conjunction with bevacizumab was notably higher at 92.3%. Preliminary findings from the Phase III COMPASSION-16 trial (NCT04982237) were disclosed in October 2024, involving 445 patients diagnosed with chronic, recurrent, or metastatic cervical carcinoma. Participants were randomly allocated into two groups (1:1), receiving either platinum-based chemotherapy in conjunction with cadonilimab or a placebo, with or without bevacizumab. The incorporation of cadonilimab resulted in a notable enhancement in PFS (12.7 months compared to 8.1 months) and OS (median not reached versus 22.8 months) [95]. However, in individuals exhibiting PD-L1 CPS values below 1, the observed advantages regarding PFS and OS did not reach statistical significance. In 2022, cadonilimab received approval for advertising in China, marking it as the inaugural bispecific antibody for immunotherapy. A notable strength of the COMPASSION-16 trial is its double-blind, placebo-controlled design, which minimizes performance and detection bias. However, the trial’s enrolled population was exclusively from China. While the high homogeneity of the population (primarily Asian) enhances internal validity, it may limit the generalizability of the results to other ethnic and regional populations. Furthermore, cadonilimab, as a bispecific antibody, has a unique toxicity profile (e.g., potentially overlapping irAEs due to simultaneous targeting of PD-1 and CTLA-4) that requires ongoing evaluation in broader patient populations and with longer follow-up durations. 

##### TIGIT Inhibitors

In addition to CTLA-4 and PD-1, TIGIT represents another inhibitory checkpoint receptor that plays a role in constraining the survival and functionality of effector T cells. TIGIT exhibits distinct functional and structural characteristics when compared to CTLA-4 and PD-1 [96]. However, the research results did not seem promising, and the relevant clinical trials were terminated three months ago.

#### 4.2.5. Combination Therapy with Immune Checkpoint Inhibitors

The demonstrated benefits of using single ICIs as first- and second-line treatment regimens have prompted investigation into the effectiveness of dual ICI regimens.

The single-arm Phase I/II 358 trial assessed the effectiveness of the anti-PD-1 antibody nivolumab, either as a monotherapy or in conjunction with the anti-CTLA-4 antibody ipilimumab, in a cohort of 176 individuals diagnosed with recurrent or metastatic cervical carcinoma, irrespective of their PD-L1 status [72,97]. Depending on the different drug doses, the ORR in the combination group ranged from 31% to 40%, while the ORR for nivolumab monotherapy was 26%.

The Phase II SKYSCRAPER-04 trial evaluated the efficacy of atezolizumab combined with the anti-TIGIT antibody tiragolumab in patients with PD-L1-positive cervical cancer (TAP ≥ 5%) receiving second- or third-line treatment [82]. The overall response rate (ORR) for the 126 patients treated with the combination regimen was 19.0%, compared with 15.6% for the 45 patients treated with atezolizumab monotherapy. In patients with a tumor proportion (TAP) ≥10%, both regimens demonstrated higher ORRs, while in patients with a TAP <10%, both regimens demonstrated lower ORRs. However, compared with historical reference data, these ORRs did not reach the predefined statistical significance threshold.

An open-label, single-arm, global Phase II clinical trial investigated the safety and efficacy of the combination therapy of balstilimab (anti-PD-1 antibody) and zimberelimab (anti-PD-1 antibody) in patients with advanced cervical cancer [73]. A total of 155 patients were included, with a median follow-up time of 21 months. An ORR of 32.6% was achieved in squamous cell carcinoma patients, with an overall disease control rate of 52%, a 12-month PFS of 21.3%, and OS rates of 53.3%. However, the median PFS was 2.7 months and the median OS was 12.8 months. The currently reported data demonstrate promising and durable clinical activity with good tolerability. At present, further studies on the combination of balstilimab (anti-PD-1 antibody) and zimberelimab are ongoing [73].

Furthermore, a randomized, double-blind, placebo-controlled Phase II trial (ClinicalTrials.gov Identifier: NCT04590599) allocated 205 patients to receive either ipilimumab plus sintilimab (*n* = 103) or placebo plus sintilimab (*n* = 102). The median PFS values for the ipilimumab–sintilimab and placebo–sintilimab groups were 3.6 months and 4.2 months, respectively. The median OS was 13.9 months in the ipilimumab–sintilimab group and 17.2 months in the placebo–sintilimab group. Based on the current clinical trial data, dual blockade with CTLA-4 and PD-1/PD-L1 yielded no significant improvement in clinical efficacy compared with monotherapy PD-1/PD-L1 blockade for recurrent metastatic cervical cancer [98].

### 4.3. Immunotherapy Combination Therapy

Pembrolizumab has shown effectiveness in individuals with chronic, persistent, or metastatic cervical malignancy. In a meticulously designed, randomized, double-blind, placebo-controlled Phase 3 KEYNOTE-A18 clinical investigation, a total of 1060 participants were systematically allocated to therapy groups, with 529 designated for the pembrolizumab–chemoradiotherapy cohort and 531 for the placebo–chemoradiotherapy cohort. The median duration of follow-up was 17.9 months, and neither cohort achieved the median progression-free survival. The survival rates at 24 months were observed to be 68% for the group receiving pembrolizumab in conjunction with chemoradiotherapy, while the group receiving placebo alongside chemoradiotherapy exhibited a rate of 57%. The overall survival rates at 24 months were observed to be 87% for the cohort receiving pembrolizumab in conjunction with chemoradiotherapy, while the cohort receiving placebo alongside chemoradiotherapy exhibited a rate of 81%. The 24-month progression-free survival rate was observed to be 68% within the pembrolizumab–chemoradiotherapy cohort, contrasting with a rate of 57% in the control cohort. The findings demonstrate that the amalgamation of pembrolizumab and chemoradiotherapy markedly enhanced progression-free survival among treatment-naive, highly susceptible individuals with locally advanced cervical carcinoma [83].

The CLAP trial represents a pioneering investigation into the concurrent inhibition of immune checkpoint and angiogenesis channels in individuals suffering from recurrent or metastatic cervical carcinoma following prior treatment interventions (Table 4). This single-arm Phase II investigation explored the incorporation of the PD-1 inhibitor camrelizumab with the selective VEGFR2 inhibitor apatinib, revealing clinical responses independent of PD-L1 expression. The patients attained an ORR of 55.6%, with an mPFS of 8.8 months and an mOS of 11.3 months [76]. A randomized, phase II trial is presently underway to assess the effectiveness of carrelizumab–apatinib in conjunction with platinum-based chemotherapy and bevacizumab for individuals suffering from recurrent metastatic cervical carcinoma. Research has investigated the integration of camrelizumab with a multi-kinase inhibitor that broadly targets receptors including VEGFR2 and 3, platelet-derived growth factor receptor beta (PDGFRβ), FMS-like tyrosine kinase-1/3 receptor, proto-oncogene tyrosine protein kinase receptor, and stem cell factor receptor. The Phase II single-arm trial demonstrated an ORR of 39.4% alongside an mPFS of 10.3 months. The median duration of follow-up was 13.6 months, with the median duration of response not yet attained, and an OS at 12 months of 77.7% [99]. The investigation into the safety and effectiveness of the amalgamation of camrelizumab and famitini for recurrent metastatic cervical carcinoma is presently underway. A Phase II clinical trial engaged 42 individuals diagnosed with recurrent or metastatic cervical carcinoma exhibiting exclusive PD-L1 expression, assessing the safety and effectiveness of the anti-PD-1 antibody sintilimab in conjunction with the multi-kinase inhibitor anlotinib. Among the 39 evaluable patients, the ORR was 59.0%. A disease control rate of 94.9% was attained, accompanied by a mPFS of 9.4 months. The median OS had not yet been reached. Therefore, the combination of sintilimab and anlotinib represents a safe and a viable treatment alternative for individuals with advanced cervical carcinoma who have not responded to previous chemotherapy [97].

## 5. Future Prospects

In recent years, significant advancements have been achieved in the management of cervical cancer, particularly for patients with locally advanced, recurrent, and metastatic disease. Driven by continuous breakthroughs in molecular profiling and tumor microenvironment analysis, the therapeutic paradigm for recurrent cervical cancer is progressively shifting from traditional radiotherapy towards precision immunotherapy and rationally designed combination therapies. Several concrete and promising research directions are emerging, poised to shape the next decade of clinical practice.

### 5.1. Refinement of Combination Strategies

Building on the success of immune checkpoint inhibitors (ICIs) with chemotherapy and bevacizumab, future efforts should focus on optimizing multi-target synergistic regimens. Key explorations include:

ICI-PARP Inhibitor Synergy: Investigating the combination of PD-1/PD-L1 inhibitors with PARP inhibitors, particularly in tumors with homologous recombination deficiency (HRD) signatures or cisplatin resistance, represents a rational strategy to enhance immunogenic cell death and overcome therapeutic resistance.

Angiogenesis-Immunity Crosstalk: Deeper exploration of the optimal sequencing and dosing of anti-angiogenic agents (e.g., bevacizumab, TKIs) with ICIs is warranted. Research should focus on modulating the immunosuppressive tumor microenvironment via vascular normalization, rather than solely on vessel destruction.

### 5.2. Targeting Novel Immune Regulatory Axes

Beyond CTLA-4 and PD-1/PD-L1, drugs targeting emerging immune checkpoints such as TIGIT, LAG-3, and TIM-3 are under active investigation. Future clinical trials should prioritize biomarker-driven patient selection (e.g., LAG-3 expression on tumor-infiltrating lymphocytes) and explore their roles in overcoming resistance to existing ICIs.

### 5.3. Leveraging Dynamic Biomarkers and AI

The development of a robust dynamic monitoring system is crucial.

ctDNA Methylation Profiling: Utilizing the methylation spectrum of circulating tumor DNA (ctDNA) not only shows promise for the early detection of recurrence risk but also enables real-time assessment of clonal evolution and treatment resistance mechanisms, guiding adaptive therapy.

AI-Driven Multi-Omics Platforms: An AI-driven platform integrating radiogenomics (features extracted from medical images), deep molecular profiling (genomics, transcriptomics), and immune infiltration characteristics could facilitate the precise screening of patients most likely to benefit from specific sequential combinations, such as PD-1 inhibitors followed by stereotactic radiotherapy.

### 5.4. Advancing Cellular Therapies and Vaccines

HPV-Targeted Therapies: TCR-T cell therapy and personalized mRNA vaccines targeting the HPV E6/E7 oncogenic proteins offer a highly specific approach to eradicate virus-driven tumor cells. Combining these with ICIs may break immune tolerance in the suppressive microenvironment.

Microenvironment Remodeling: The post-radiotherapy fibrotic niche presents a major barrier to drug delivery and immune cell infiltration. Research into matrix-targeted drugs (e.g., FAK inhibitors, collagenase) aimed at remodeling this physical barrier, combined with cellular therapies, holds significant potential.

### 5.5. Pioneering Novel Biologics

Breakthroughs in bispecific antibodies (e.g., cadonilimab), antibody-drug conjugates (ADCs), and oncolytic virus therapies are reshaping the immunotherapy landscape. Future work should focus on identifying predictive biomarkers for response, managing unique toxicity profiles, and developing rational combinations with other modalities.

Ultimately, establishing a cross-disciplinary collaborative research network that integrates radiobiology, computational medicine, and translational immunology will be paramount. This concerted effort has the potential to drive a fundamental shift in the management of recurrent cervical malignancy, moving decisively from palliative control towards the ambitious goal of functional cure.

## 6. Conclusions

The management of pelvic recurrence after radiotherapy for cervical cancer remains a formidable clinical challenge, yet significant strides have been made in expanding the therapeutic arsenal. This review has detailed the evolution from traditional local approaches, such as salvage surgery and re-irradiation, to the modern era of systemic therapy dominated by targeted agents and immunotherapy. The integration of anti-angiogenic drugs, PARP inhibitors, tyrosine kinase inhibitors, and particularly immune checkpoint inhibitors like pembrolizumab, has fundamentally improved outcomes for patients with recurrent or metastatic disease. These advancements underscore a critical shift towards personalized medicine, where treatment selection is increasingly guided by molecular characteristics and individual patient profiles.

Looking ahead, the future of treating recurrent cervical cancer lies in the strategic combination of these modalities. Multimodal regimens that synergize immunotherapy with targeted therapy, chemotherapy, and even precision radiotherapy hold immense promise for overcoming resistance mechanisms and improving survival rates. Furthermore, emerging technologies—such as bispecific antibodies, cellular therapies, circulating tumor DNA monitoring, and AI-driven biomarker platforms—are poised to further refine treatment paradigms. The ultimate goal is to transform the management of this condition from palliative control towards achieving durable remission and functional cure, offering new hope to patients facing this difficult diagnosis.

## Figures and Tables

**Table 1:** Summary of Targeted Drugs.

Drug Name	Mechanism of Action	Key Outcomes (PFS/OS)	Current Status
Bevacizumab [7]	Anti-VEGF monoclonal antibody	GOG-240: mPFS 8.2 vs. 5.9 m; mOS 16.8 vs. 13.3 m	Approved(FDA-approved for 1st-line)
Apatinib [20]	Selective VEGFR2 Tyrosine Kinase Inhibitor (TKI)	Meta-analysis (*n* = 243): mPFS 5.19 m; mOS 10.63 m	Clinical Trials (Phase II)
Pazopanib [21]	Multi-target TKI (VEGFR-1,2,3, PDGFR, c-Kit)	Phase II: mOS 50.7 w (vs. Lapatinib 39.1 w)	Not standard, needs further study
Cediranib [22]	TKI (VEGFR1–3, PDGFR-α)	Phase II: mPFS 8.1 vs. 6.7 m (with chemo vs. chemo)	Clinical Trials (Phase II)
Nintedanib [23]	TKI (VEGFR 1–3, FGFR 1–3, PDGFR-α/β)	Phase II: mOS 21.7 vs. 16.4 m (with chemo vs. chemo)	Clinical Trials (Phase II)
Sunitinib [24]	Multi-target TKI (VEGFR, PDGFR, c-Kit, FLT3)	Phase II (*n* = 16): mPFS 3.5 m	Inconclusive evidence, needs further study
Tisotumab Vedotin [25]	Tissue Factor-targeting Antibody-Drug Conjugate (ADC)	innovaTV 301 Phase III: mOS 11.5 vs. 9.5 m; mPFS 4.2 vs. 2.9 m	Approved(FDA-approved for 2nd-line)
Nimotuzumab [26]	Anti-EGFR monoclonal antibody	Multiple studies showed improved CR rate and PFS when combined with chemoradiation	Clinical Trials (primarily in China)
Cetuximab [27]	Anti-EGFR monoclonal antibody	Phase II: No significant improvement in PFS or OS when added to chemotherapy	Trial endpoint not met, not standard
Gefitinib [28]	EGFR Tyrosine Kinase Inhibitor (TKI)	Studies showed limited efficacy as monotherapy	Under investigation, potential for combination
Olaparib [29]	PARP Inhibitor	Preclinical and case report data show efficacy; associated with cisplatin resistance	Preclinical/Early Clinical Trials (Phase I/II)
Veliparib	PARP Inhibitor	Phase I/II: ORR 60%, mPFS 6.2 m, mOS 14.5 m (with chemo)	Clinical Trials (Phase I/II)
Rucaparib [30]	PARP Inhibitor	Phase II: ORR of 14% in cervical cancer when combined with bevacizumab	Approved(FDA-approved for maintenance therapy)

m: month; w: week.

**Table 2:** Summary of Antibody-Drug Conjugates.

Target	Agent	Stage	Studies
TF	TV [25]	Recurrent and metastatic cervical cancer with disease progression on or after chemotherapy	NCT03438396 NCT04697628 NCT03786081
HER2	T-DXd [53]	Unresectable or metastatic HER2positive solid tumors	NCT04482309 NCT04639219
	IBI354 [54]	Solid tumors	NCT05636215
Trop-2	SG [55,56]	Metastatic TNBC, HR+/HER2BC	NCT05119907
	SKB264 [57]	Locally advanced or metastatic TNBC and EGFR mutationpositive nonsquamous NSCLC after 2L systemic therapy	NCT05642780 NCT06459180
Mesothelin	RC88 [58]	Solid tumors	NCT04175847
Nectin-4	9MW-2821 [59]	Solid tumors	NCT05216965

+: positive.

**Table 3:** Immunotherapy in cervical cancer.

Studies	Agent	Target	Stage
NCT03972722 [72]	Zimberelimab	Anti-PD-1	Recurrent or metastatic cervical cancer
NCT03676959 [73]	Socazolimab	Anti-PD-L1	Recurrent or metastatic cervical cancer
NCT04886700 [74]	Enlonstobart	Anti-PD-1	PD-L1+ Recurrent or metastatic cervical cancer
NCT03852823 [75]	Enlonstobart	Anti-PD-1	Recurrent or metastatic cervical cancer
NCT03852251 [76]	Cadonilimab	Anti-PD-1 and CTLA-4	Recurrent or metastatic cervical cancer
NCT05557565	Iparomlimab and Tuvonralimab	Anti-PD-1 and CTLA-4	Recurrent or metastatic cervical cancer
NCT02054806 [77]	Pembrolizumab	Anti-PD-1	PD-L1+ Recurrent or metastatic cervical cancer
NCT02628067 [78]	Pembrolizumab	Anti-PD-1	Recurrent or metastatic cervical cancer
NCT02488759 [79]	Nivolumab	Anti-PD-1	Recurrent or metastatic cervical cancer

**Table 4:** Combination treatment regimens.

Combination Regimen	Trial Name/Type	Median PFS (Months)	Median OS (Months)	Other Survival Outcomes
Pembrolizumab + Chemotherapy (±Bevacizumab)	KEYNOTE-826 (Phase III)	-	26.4	28.6 (CPS ≥ 1); 29.6 (CPS ≥ 10
Pembrolizumab + Chemoradiotherapy	KEYNOTE-A18 (Phase III)	24 m PFS Rate: 68%	24 m OS Rate: 87%	Control: 57% PFS, 81% OS
Atezolizumab + Chemotherapy + Bevacizumab	BEATcc (Phase III)	13.7	32.1	Control: 10.4 PFS, 22.8 OS
Cadonilimab + Chemotherapy (±Bevacizumab)	COMPASSION-16 (Phase III)	12.7	Not Reached	Control: 8.1 PFS, 22.8 OS
Camrelizumab + Apatinib	CLAP (Phase II)	8.8	11.3	ORR 55.6%
Sintilimab + Anlotinib	Phase II	9.4	Not Reached	ORR 59.0%
Nivolumab + Chemoradiotherapy	NiCOL (Phase I)	2-y PFS Rate: 75%	-	ORR 93.8%, CR 50%

## Abbreviations

OS	overall survival
mOS	median overall survival
PFS	progression free survival
mPFS	progression free survival
DoR	duration of response
mDoR	median duration of response
SD	stable disease
DCR	disease control rate
ORR	objective response rate
CRR	complete response rate
HPV	human papilloma virus
CPS	combined positive score
EGF	epidermal growth factor
VEGF	vascular endothelial growth factor
EGFR	epidermal growth factor receptor
VEGFR	vascular endothelial growth factor receptor
PAR	poly ADP-ribose
PARP	poly ADP-ribose polymerase
FDA	Food and Drug Administration
ATP	adenosine triphosphate
TKI	tyrosine kinase inhibitor
ADC	antibody-drug conjugate
CTLA-4	cytotoxic T lymphocyte antigen 4
PD-L1	programmed cell death protein 1
ITIM	immune receptor tyrosine-based inhibitory motif
TIGIT	T cell immunoglobulin and ITIM domains
LAG-3	lymphocyte activation gene 3
ICI	immune checkpoint inhibitor
TAP	tumor area positivity score
